# Supplementary material for: Axonal Transport as an In Vivo Biomarker for Retinal Neuropathy
Source: Cells. 2020 May 22;9(5):1298. doi: 10.3390/cells9051298 (PMC7291064; doi:10.3390/cells9051298)
Supplement: Supplementary file 1 [file cells-09-01298-s001.pdf]

Supplemental Materials

Supplemental Table 1: The allocation of the number of animals per experiment.

Experiments	Animal number
Initial TTc-488 probe dose optimization and autofluorescence determination	5
Initial immunofluorescence optimization for anti RBPMS and anti-SMI32	5
In vivo retinal ophthalmoscopy time course imaging for TTc-488 (Fig 1)	3
Immunofluorescence with anti-RBPMS (Fig. 1)	3
Ex vivo retinal ROI (Fig. 2) and transect analysis (Fig. 3)	5
Immunofluorescence with anti-SMI32 for co-localization studies (Fig. 4)	4
Individual retinal synaptic layer thickness (Fig. 5)	5

Supplemental Table 2: Confocal imaging laser settings.

**Immunofluorescence with anti-SMI32 for co-localization studies (Fig. 4).**	
488 nm PMT voltage	395 V
543 nm PMT voltage	482 V
633 nm PMT voltage	389 V
488 nm laser transmittivity	10%
543 nm PMT transmittivity	50%
**Individual retinal synaptic layer thickness (Fig. 5)**	
488 nm PMT voltage	425 V
543 nm PMT voltage	482 V
633 nm PMT voltage	389 V
488 nm laser transmittivity	10%
543 nm PMT transmittivity	50%
633 nm PMT transmittivity	10%

PMT -photomultiplier tube

Supplemental Diagram 1: The experimental set-up for the NMDA treatment and TTc-488 neural probe injection scheme.

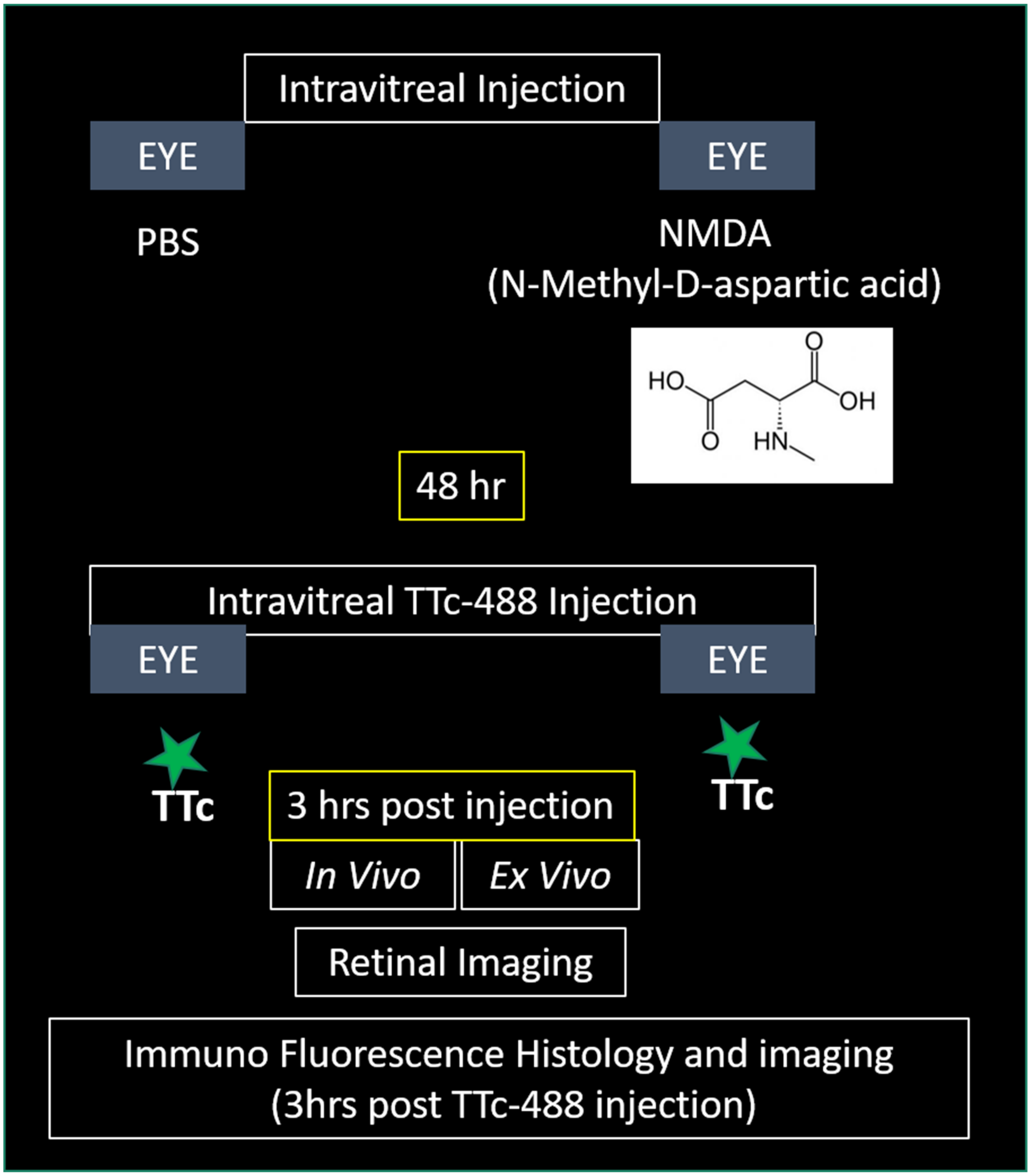

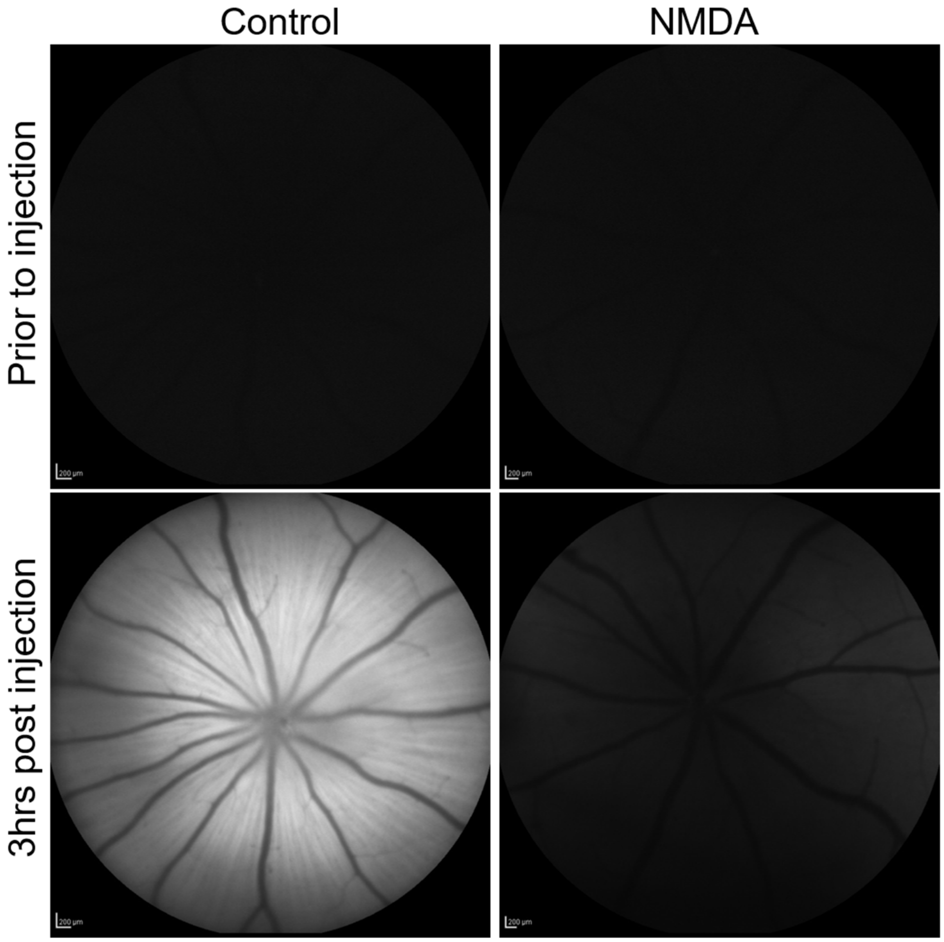

Supplemental Figure 1: Autofluorescence readings for *in vivo* CSLO imaging prior to and after a 3hr TTc-488 intravitreal injection. The average pre-injection fluorescent intensity values for control (16.08 AU ± 0.89) vs. NMDA treated eyes (16.20 ± 0.76) in comparison with the fluorescence intensity of TTc-488, 3hrs after injection, for control (141.67 AU ± 4.65) vs. NMDA treated eyes (31.28 ± 23.18) (Mean ±SD). The baseline autofluorescence values is substantially lower for the pre-injected eyes in comparison with the TTc-488 injected eyes as expected. Scale bar 200 µm.

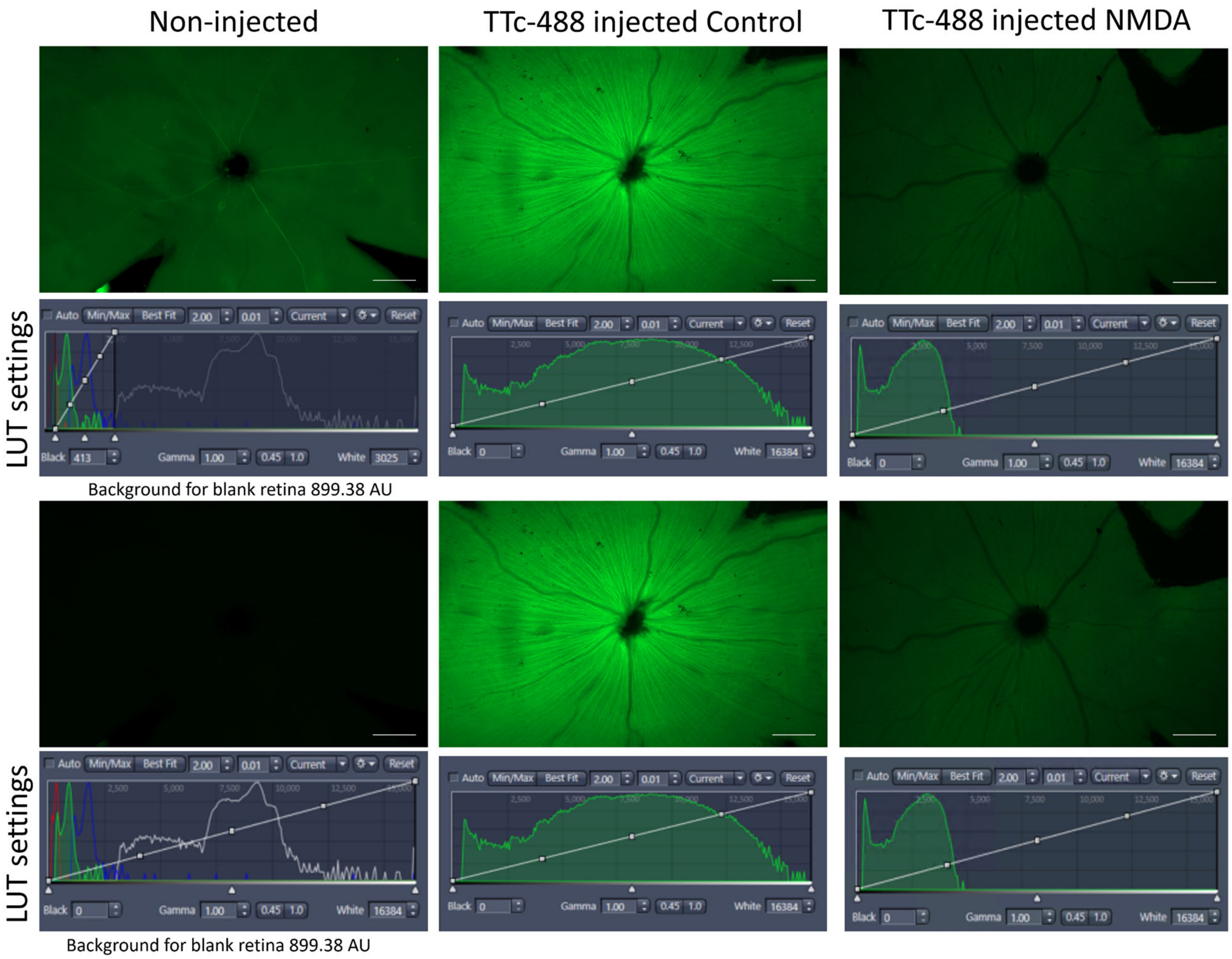

Supplemental Figure 2: Autofluorescence readings for *ex vivo* retinal flat mount imaging with widefield fluorescence microscopy (Zeiss AZ16) and ROI analysis. The measure of the average fluorescence intensity for the non-injected retina was 899.38 AU and changing the LUT min and max values did not change the outcome of fluorescence intensity readings (compare top and bottom panels). The gamma output was kept constant during analysis. The average fluorescence intensities for the control (6185.09 AU ± 2262.34) vs. NMDA (2626.68 AU ± 1860.79) treated eyes were significantly different from each other and obviously different from the background values for the blank non-injected retina. Histogram x-axis scaling was changed in the top panel to make the retina of the non-injected eye visible while the histogram x-axis scaling for the bottom panel was held constant to facilitate true background comparisons. Scale bars 500µm.

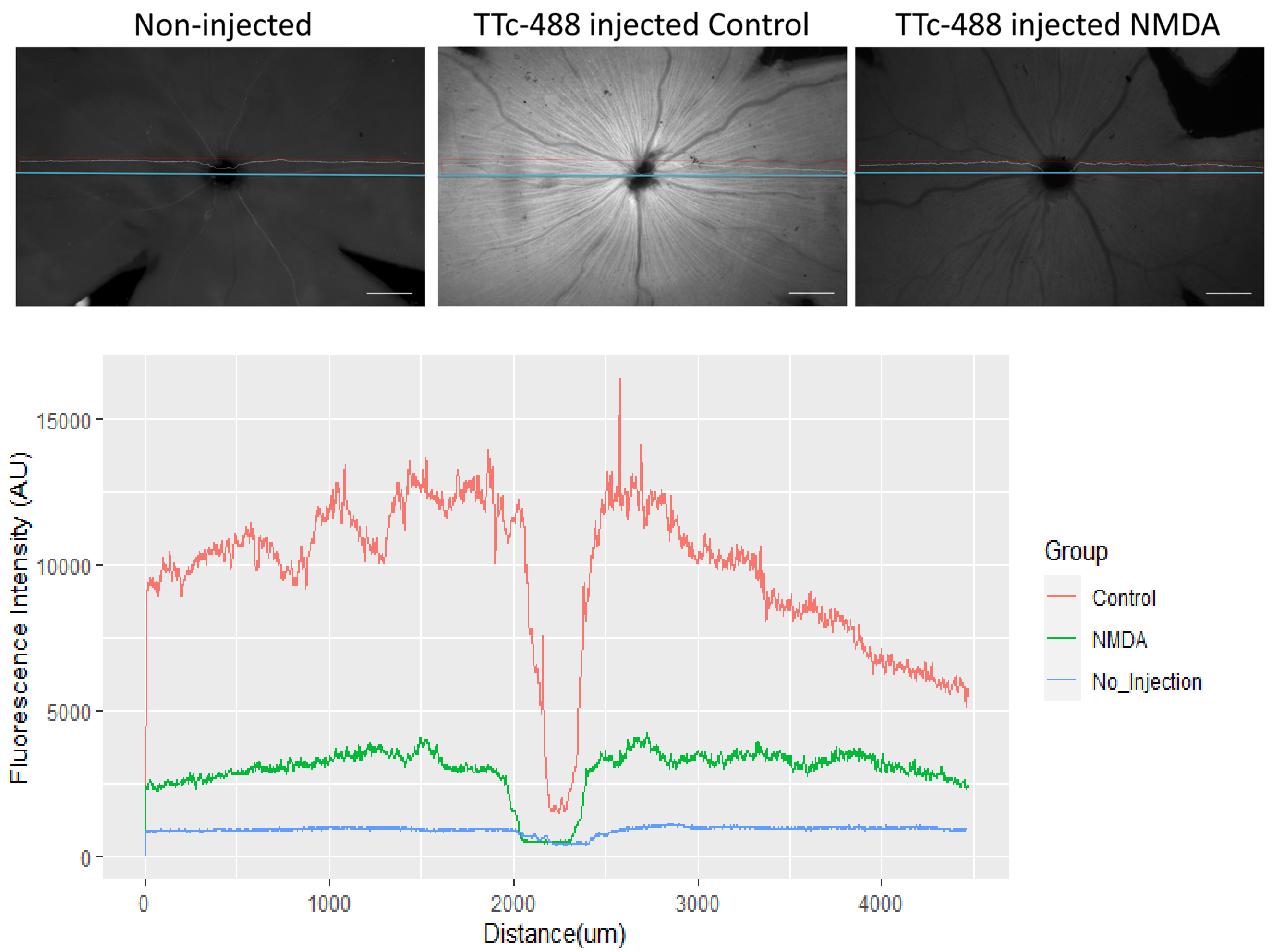

Supplemental Figure 3: Autofluorescence readings for *ex vivo* retinal flat mount imaging with widefield fluorescence microscopy (Zeiss AZ16) and retinal transect analysis. A linear retinal transect through the middle of the optical nerve head (blue line) was used to demonstrate the differences in fluorescence intensity between non-injected, control and NMDA-treated retinas. There is an obvious difference between the fluorescence intensity of non-injected eyes in comparison with TTc-488 injected control and NMDA treated eyes. The sharp decline in fluorescence intensity between 2000-2500 µm distance, for all groups, correspond to the area in the retinal flat mount where the optical nerve head was removed during dissection. Scale bars 500 µm.
